# Methodology for Evaluating Behavior of Reinforced Concrete Slabs in Temporary Traffic Bridge Systems over Uncured Cement Concrete Pavements Using Small-Scale Experimental Slabs

**DOI:** 10.3390/ma19071302

**Published:** 2026-03-25

**Authors:** Soon Ho Baek, Kang In Lee, Sang Jin Kim, Geon Lee, Seong-Min Kim

**Affiliations:** Department of Civil Engineering, Kyung Hee University, Yongin 17104, Republic of Korea; qortnsgh1@khu.ac.kr (S.H.B.); leerkddls123@khu.ac.kr (K.I.L.); kai7290@khu.ac.kr (S.J.K.); aes3334@khu.ac.kr (G.L.)

**Keywords:** reinforced concrete, slab, small scale, full scale, reduction ratio, temporary traffic bridge system, cement concrete pavement

## Abstract

A methodology was developed to evaluate the behavior of reinforced concrete slabs used in temporary traffic bridge systems installed over uncured cement concrete pavement sections using highly scaled-down experimental reinforced concrete slabs. A full-scale reinforced concrete slab was first designed and its behavior, such as strain and deflection, was numerically analyzed. A small-scale reinforced concrete slab was then designed considering a dimensional reduction ratio of 1/6. When using this reduction ratio, there is no actual reduced size steel bar, so the smallest size steel bar available must be used for placement. Therefore, numerical analyses were performed to design the steel bar arrangement of the small-scale slab so that the same behavior as that of the full-scale slab occurred. To conduct experiments, small-scale experimental slabs were fabricated according to the design. Since the size of coarse aggregates must be reduced in concrete used for small-scale slabs, specimens using the concrete mix design for full-scale slabs were also produced and the compressive strengths were compared to confirm that the strengths were the same. Next, a study was conducted on the selection of strain gauges that can be used in small-scale slab experiments, and a method for installing displacement gauges to accurately measure slab deflection was also designed. Based on this series of basic studies, load tests were performed to measure the strains and deflections of small-scale slabs. Comparing the measured behavior of the small-scale slab with the numerical analysis results, it was confirmed that the same behavior was observed. Therefore, the experimental results and numerical analysis results of the small-scale slab were consistent, and the numerical analysis results of the small-scale slab and the full-scale slab were identical, proving that the experimental results of the full-scale slab can be inferred through experiments using the small-scale slab. This study confirmed that if small-scale slabs are designed and manufactured to appropriately reflect the characteristics of full-scale slabs, even though the process is challenging, the behavior of full-scale slabs can be approximately determined through experiments using small-scale slabs.

## 1. Introduction

Traffic volume in urban areas continues to increase due to increasing population density and expanding logistics demand. In particular, since most urban roads in Korea are paved with asphalt, early distresses frequently occur due to the combined effects of repetitive acceleration and deceleration loads from heavy vehicles and environmental loads caused by climate change [1,2,3,4]. The resulting frequent maintenance entails massive budget consumption and causes severe public inconvenience due to traffic closures during rehabilitation [5,6]. As an alternative to overcome these problems, using highly durable cement concrete pavements can be an excellent solution. However, the essentially required curing time and the subsequent traffic control are the most significant practical constraints for their widespread application in urban areas. Although construction and curing times can be shortened by utilizing precast slabs [7,8,9,10,11,12,13,14,15,16] or rapid-hardening cement concrete [17,18,19,20,21,22,23,24,25], their widespread implementation is limited in terms of economic feasibility due to high construction costs.

**Figure 1:**
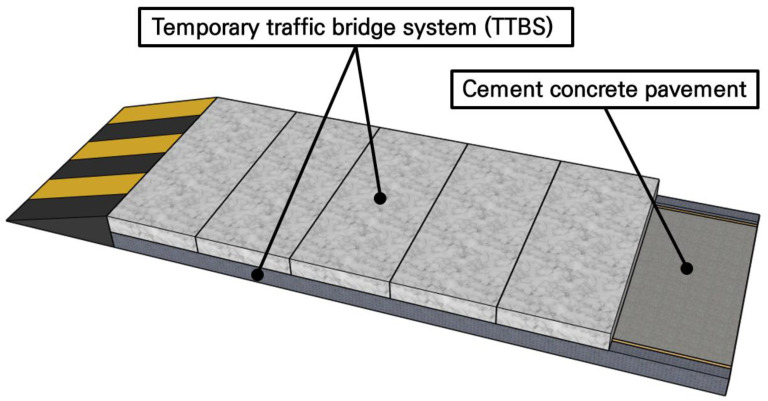
Conceptual diagram of TTBS.

To resolve these issues, an innovative temporary traffic bridge system (TTBS) that can be installed temporarily to allow immediate traffic opening while the concrete pavement cures has been designed by our research team [26,27]. While various materials can be used to manufacture the TTBS, research was conducted primarily on utilizing concrete slabs as the main members due to their outstanding economic efficiency and durability [28,29]. Figure 1 shows a conceptual diagram of the concrete slab-based TTBS. First, the existing pavement is removed, formwork is installed, and a cement concrete pavement is placed. Then, the TTBS slabs are installed on the support blocks for immediate traffic opening through the TTBS during the curing period of the cement concrete pavement. Once the cement concrete pavement is fully cured, the TTBS slabs are removed and traffic is allowed through the cement concrete pavement. For the successful field application of the TTBS, safety verification of the slab directly supporting the loads is essential. However, full-scale slab fabrication and load testing entail significant practical difficulties, including limitations in cost, space, and equipment. Therefore, the development of a highly reliable small-scale test methodology capable of accurately representing the results of full-scale tests is needed.

As an alternative to such full-scale tests, studies on small-scale tests have often been conducted in which actual structures are scaled down to a certain ratio. Some studies report the results of analyzing the behavior of structures under various loading conditions using various dimensional reduction ratios ranging from 1/2 to 1/10 [30,31,32,33]. However, previous studies using highly reduced models were mostly limited to plain concrete slabs or simple structures [34,35], and for structures containing reinforcing bars, small-scale models were mainly limited to relatively low-dimensional reduction ratios, such as 1/2 or 1/4 [36,37,38,39,40,41,42,43,44,45,46,47,48]. This is because applying a high-dimensional reduction ratio to reinforced concrete members entails complex physical constraints that are difficult to overcome through simple geometric scaling alone. These include the difficulty in obtaining equivalent reinforcement ratios due to limitations in the size of commercially available steel bars, concrete strength degradation caused by the reduction in the maximum aggregate size, and measurement limitations associated with monitoring micro-deformations. Thus, it is essential to establish a systematic design and verification procedure for very-small-scale structures capable of securing behavioral equivalence with full-scale structures by considering material properties.

The objective of this study is to develop and validate a methodology for reliably predicting the behavior of the TTBS slabs applied to curing concrete pavement sections through the testing using 1/6 scale reinforced concrete slabs. Through this study, the structural safety of the TTBS slabs can be evaluated economically and efficiently, and the proposed methodology can also be used to predict the behavior of other reinforced concrete structures under various loading conditions. To achieve the purpose of this study, the optimal reinforcement arrangement and concrete mix designs for small-scale slabs, capable of identically simulating the behavior of full-scale slabs, were first derived through numerical analysis, and specimens were fabricated based on this. Subsequently, load testing was performed on the small-scale experimental slabs to evaluate their structural behaviors. Finally, the validity of the proposed behavior prediction methodology was proven by cross-validating the experimental results with small-scale and full-scale numerical analysis results. This paper describes these research processes and results in detail.

## 2. Research Methodology

**Figure 2:**
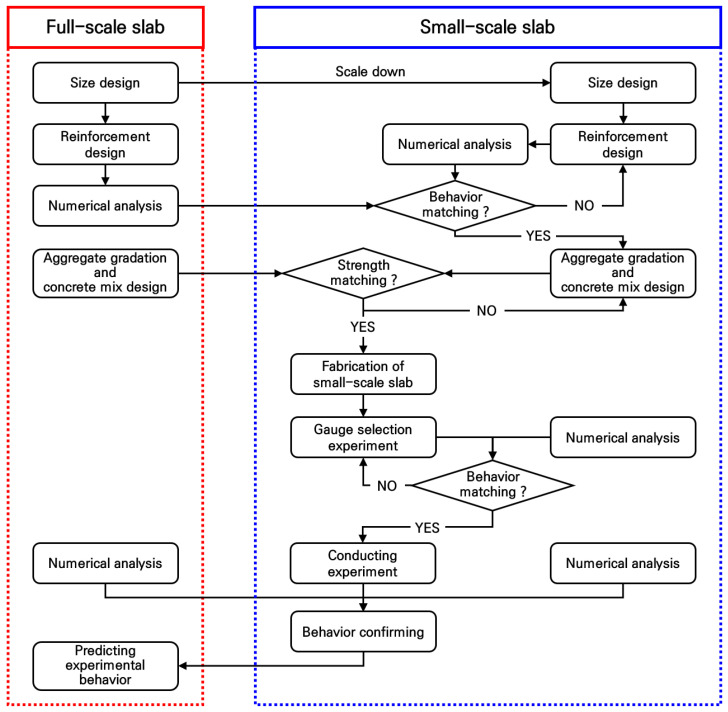
Research methodology.

In this study, a methodology is proposed to predict the behavior of full-scale reinforced concrete slabs through experiments using small-scale reinforced concrete slabs with a high-dimensional reduction ratio of 1/6. The reason for using a reduction ratio of 1/6 was to account for the effective width of experimental equipment such as UTM. The research is conducted in four steps as described below, and Figure 2 summarizes this research methodology.

In the first step, a full-scale reinforced concrete slab used for temporary traffic bridge systems is designed in terms of size, reinforcement arrangement, and concrete mix in accordance with the intended use and concrete structure design standards. The designed full-scale slab is evaluated for its structural stability by evaluating its behavior, such as strain and deflection, under vehicle load conditions through numerical analysis, and this behavior is used as a reference behavior when designing a small-scale reinforced concrete slab.

In the second step, a small-scale slab with a 1/6 reduction ratio is designed that exhibits structurally identical behavior to the full-scale slab. For steel bars that cannot be reduced according to the reduction ratio, the optimal reinforcement arrangement design is performed through numerical analysis to obtain stresses equivalent to those of a full-scale slab using the commercially available minimum size of steel bars. In addition, to prevent changes in the mechanical properties of small-scale slabs due to a decrease in aggregate size, concrete mix design is performed by adjusting the aggregate size, and after confirming that the concrete material is equivalent to that of a full-scale slab through a compressive strength test, small-scale experimental slabs are finally fabricated.

There may be a question whether the length of the strain gauges used in full-scale experiments must also be reduced to match the reduction ratio when conducting small-scale experiments. In the third step, the optimal strain gauges are selected by considering inhomogeneous characteristics of the concrete material in order to precisely measure the behavior of the fabricated small-scale experimental slabs. Additionally, a deflection measurement system is designed to compensate for the influence of micro-displacements of the structure supporting the small-scale slab on the deflection measurement of the slab. Afterwards, a load test is performed on a small-scale slab, and the results are compared with the numerical analysis results to verify the mutual validity of the experiment and numerical analysis.

In the final step, the experimental and numerical analysis results of the small-scale slab, which show the same values, are compared with the numerical analysis results of the full-scale slab. In this case, if the experimental and numerical analysis results of the small-scale slab are identical to the numerical analysis results of the full-scale slab, it can be expected that the same experimental results will be obtained when conducting experiments on the full-scale slab. This process demonstrates that small-scale reinforced concrete slabs can be used to conveniently and economically predict the experimental results of full-scale reinforced concrete slabs. Moreover, if a larger number of small-scale slabs are fabricated and tested, statistical indicators such as the standard deviation and the coefficient of variation could be obtained, thereby enabling a more rigorous assessment of the reliability and variability of the predicted full-scale slab behavior.

## 3. Design and Numerical Analysis of Full-Scale Slab

### 3.1. Design

The design procedure for the full-scale slab was systematically conducted in the order of dimension, reinforcement, aggregate gradation, and concrete mix designs. In the dimension design phase, it was considered that the slab used in this study should function as a temporary traffic bridge system. Accordingly, the width of the slab was selected as 3.48 m to accommodate the general road lane width of 3 m or more and to consider the 0.18 m wide supports at both ends. The length of the slab was selected as 1.5 m considering constructability and transport efficiency. In addition, the thickness of the slab was designed to be 0.3 m, which is a typical thickness for concrete pavement slabs with verified structural stability.

The reinforcement design complied with the Korean Design Standards (KDS), including KDS 14 20 50 (Detailed design standards for concrete structure reinforcement) [49], KDS 14 20 01 (General requirements for concrete structure strength design method) [50], and KDS 14 20 10 (Analysis and design principles) [51]. As major design variables, the unit weight of concrete was applied as 22.6 kN/m^3^, the specified compressive strength as 30 MPa, and the flexural strength reduction factor as 0.85. The D16 (16 mm diameter) deformed steel bars, the most commonly used in slab design, were used. To calculate the bending moment acting on the full-scale slab, the support conditions were configured as an overhanging beam near both transverse ends, considering the situation where supports are installed at the bottom of the slab.

**Figure 3:**
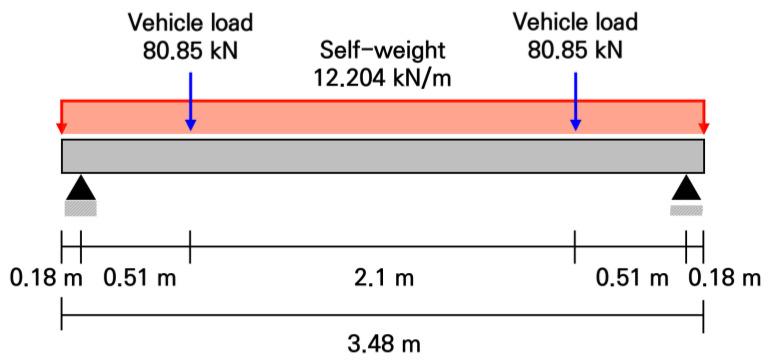
Loading conditions on full-scale slab.

The loads were analyzed by dividing them into the self-weight of the slab and the vehicle loads. When calculating the self-weight, the total self-weight was calculated as 35.392 kN by multiplying the total concrete volume of the slab by the unit weight, and the design self-weight was derived by applying a load factor of 1.2. As shown in Figure 3, assuming the self-weight of the slab as a uniformly distributed load, the bending moment due to the self-weight was calculated to be 14.652 kN·m. For the vehicle loads, it was assumed that a heavy vehicle, such as a bus, moves over the slab. To consider the most unfavorable loading condition, the rear axle load was calculated to be 101.063 kN by applying a rear axle weight distribution ratio of 62.5% to the fully loaded weight of a two-axle city bus, which is 161.7 kN [52]. The design vehicle wheel load was calculated as 80.85 kN by multiplying a load factor of 1.6 to the wheel load of 50.532 kN, which was distributed to each wheel. As shown in Figure 3, considering the distance between the wheels, the wheel loads were applied at locations 0.69 m away from both ends of the slab. As a result, the bending moment due to the vehicle loads was calculated to be 41.234 kN·m. Finally, the total required bending moment, simultaneously considering the self-weight of the slab and the vehicle loads, was derived a s 55.886 kN·m, as given in Equations (1)–(3).

(1) $$M_{gravity}=\frac{\omega L^{2}}{8}-\frac{\omega a^{2}}{2}=\frac{12.204\;kN/m\times {\left(3.12\;m\right)}^{2}}{8}-\frac{12.204\;kN/m\times {\left(0.18\;m\right)}^{2}}{2}=14.652\;kN\cdot m$$

(2) $$M_{vehicle}=1.6P\times b=\left(1.6\times 50.532\;kN\right)\times 0.51\;m=41.234\;kN\cdot m$$

(3) $$M_{total}=M_{vehicle}+M_{gravity}=41.234\;kN\cdot m+14.652\;kN\cdot m=55.886\;kN\cdot m$$

In Equation (1), $M_{gravity}$ is the bending moment due to the self-weight, *w* is the self-weight/m, *L* is the length between the supports, and *a* is the overhang length. In Equation (2), $M_{vehicle}$ is the bending moment due to the vehicle loads, *P* is a wheel load, and *b* is the distance to the wheel load from the support. In Equation (3), $M_{total}$ is the total bending moment, simultaneously considering the self-weight of the slab and the vehicle loads.

To evaluate the crack resistance of the cross-section based on the modulus of rupture of the concrete, the cracking moment was calculated to be 77.65 kN·m, as given in Equations (4) and (5). By comparing the cracking moment with the total bending moment resulting from the sum of the self-weight and vehicle loads, it was found that the cracking moment exceeded the total bending moment. Accordingly, to prevent brittle failure of the slab and to ensure sufficient structural safety, the design flexural strength was re-established based on the cracking moment. Specifically, a strength reduction factor was applied by comprehensively considering the quality variation of concrete materials, manufacturing errors of the cross-sectional shape, and uncertainties in the construction process. Through this, the minimum required flexural strength was calculated as 109.624 kN·m, as given in Equation (6). It should be noted that reinforced concrete slabs were considered in this study even under small loads because in reinforced concrete slabs, even before cracks occur, the behaviors such as strain and displacement differ from those of plain concrete slabs depending on the amount and arrangement of reinforcement.

(4) $$fr=0.63\sqrt{f_{ck}}=0.63\sqrt{30\;MPa}=3.451\;MPa=3451\;kN/m^{2}$$

(5) $$M_{cr}=fr\times \frac{d\times h^{2}}{6}=3451\;kN/m^{2}\times \frac{1.5\;m\times {\left(0.3\;m\right)}^{2}}{6}=77.65\;kN\cdot m$$

(6) $$\Phi \times M_{n}\geq 1.2M_{cr}\;\to M_{n}\geq \frac{1.2M_{cr}}{\Phi }=\frac{1.2\times 77.65\;kN\cdot m}{0.85}=109.624\;kN\cdot m$$

In Equation (4), fr is the modulus of rupture and $f_{ck}$ is the design compressive strength of concrete. In Equation (5), $M_{cr}$ is the cracking moment, *d* is the longitudinal length of the slab, and *h* is the slab thickness. In Equation (6), Φ is the flexural strength reduction factor and $M_{n}$ is the minimum required flexural strength.

A detailed reinforcement design was performed to satisfy the required strength. To ensure durability, the concrete cover thickness, a major design factor, was set to 75 mm, which is larger than the minimum standard of 50 mm for precast concrete in KDS 14 20 50 [49]. The slab thickness was set to 300 mm through the dimension design. The diameter of the main reinforcement was selected as 16 mm (D16), and based on the determined cross-sectional dimensions and effective depth, the required reinforcement ratio for the slab was calculated to be 0.379%. To satisfy this, a total of 7 transverse reinforcements using D16 deformed steel bars were arranged with a spacing of 225 mm. To control cracking caused by temperature changes and drying shrinkage, the longitudinal reinforcement was designed by applying the minimum steel ratio of 0.2% recommended by Korean guidelines [49]. Regarding the actual treatment of the steel bar ends, it is pointed out that sufficient anchorage length can be secured by bending the steel bar ends at a 90-degree angle, but in this study, it was assumed that the steel bar ends were simply cut without considering actual steel bar placement details such as the treatment of the steel bar ends. The final reinforcement design details of the full-scale slab are shown in Table 1.

The aggregate gradation and concrete mix designs of the full-scale slab were performed in compliance with the Korean cement concrete pavement construction guidelines [53]. In the aggregate gradation design, crushed granite was used and the maximum sizes of coarse and fine aggregates were selected as 20 mm and 10 mm, respectively. The overall gradation distribution was adjusted to be close to the median gradation of the specified upper and lower limit curves. In the concrete mix design, to ensure the durability of the concrete, the water-cement ratio was adjusted to 40%, which is lower than the standard value of 45%, and the fine aggregate ratio was set to 49%. Based on this, the unit water content, unit cement content, and aggregate content were calculated, and the finally obtained aggregate gradation and concrete mix design results are shown in Table 2.

### 3.2. Numerical Analysis

**Figure 4:**
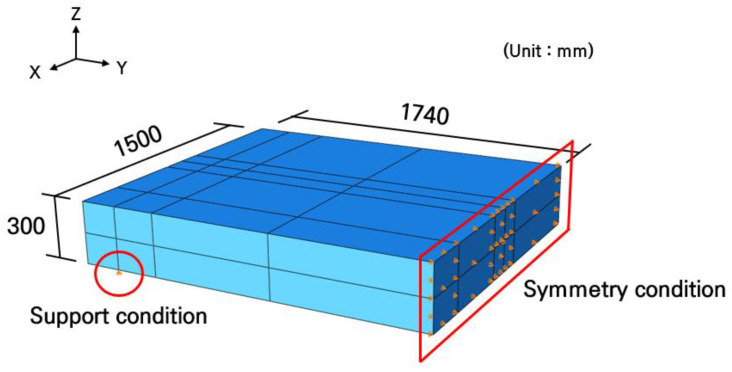
Full-scale slab analysis model.

To evaluate the structural safety and behavior of the full-scale slab under vehicle loading conditions, a three-dimensional finite element analysis was performed using ABAQUS 2024 [54] software, and the analysis model was created as shown in Figure 4. Although the overall dimensions of the slab are 1500 mm in length, 3480 mm in width, and 300 mm in thickness, a symmetric model with the width halved to 1740 mm was applied to improve the analysis efficiency, considering the lateral symmetry of the geometric shape.

In the analysis, concrete and steel bars were assumed to be linear elastic materials. The concrete slab was modeled using 8-noded solid elements, with an elastic modulus of 27.5 GPa and a Poisson’s ratio of 0.15. The elastic modulus of C30 concrete may also vary depending on the adopted design standards. Eurocode specifies an elastic modulus of about 33 GPa for C30 concrete [55], whereas the Korean design code provides a value of about 27.5 GPa [56]. Therefore, in this study, an elastic modulus of 27.5 GPa was adopted according to Korean design standards, as it was determined to be more suitable for the material properties of Korea. The Poisson’s ratio of C30 concrete is generally considered to be about 0.20 [55], but since the Poisson’s ratio of concrete is commonly reported to range from 0.15 to 0.25 [57,58], a value of 0.15 was adopted in this study. In addition, when a Poisson’s ratio of 0.20 was applied for further analysis, the differences in strain, displacement, and maximum principal tensile stress were found to be only 0.11%, 1.26%, and 0.11%, respectively. This indicates that the variation in Poisson’s ratio has only a very limited effect on the analysis results. The longitudinal and transverse steel bars were modeled using wire elements and arranged identically to the reinforcement design. The elastic modulus and Poisson’s ratio of the steel bars were set to 200 GPa and 0.3, respectively. It was assumed that the steel bars and concrete were perfectly bonded because the analysis was performed in the linear elastic region of the material as previously noted. The complete bonding between the steel bars and concrete was simulated using the embedded region constraint technique.

For the boundary conditions, the vertical displacements of the reinforced concrete slab along the longitudinal direction at the support locations were constrained to simulate the overhanging slab behavior caused by the supports. In addition, the transverse displacements of the reinforced concrete slab along the longitudinal centerline were constrained to impose a symmetric boundary condition. The optimal mesh size was determined through a convergence analysis, and a uniform mesh size of 15 mm was generated for the entire domain.

**Figure 5:**
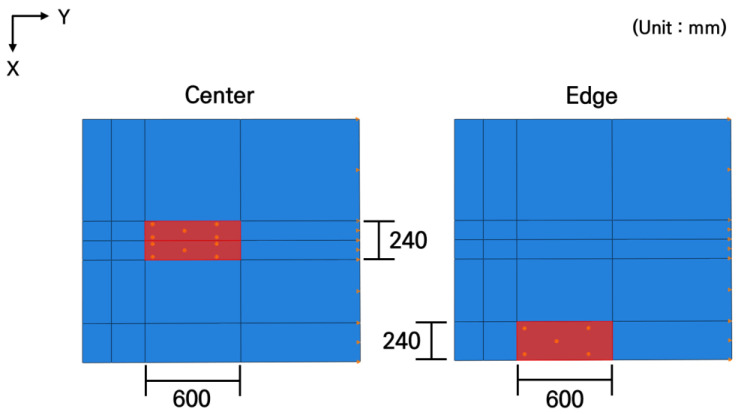
Loading locations.

The loading conditions were simulated as the center and edge loads depending on the vehicle’s moving positions, as shown in Figure 5, representing the situations where the vehicle wheels are located at the center and the edge of the slab, respectively. The transverse location of the loads was determined considering the wheelbase of the bus, and a rectangular loading area was applied, which was calculated by dividing the rear axle load of the bus by the tire pressure. As previously described, the magnitude of the load was 50.531 kN.

**Figure 6:**
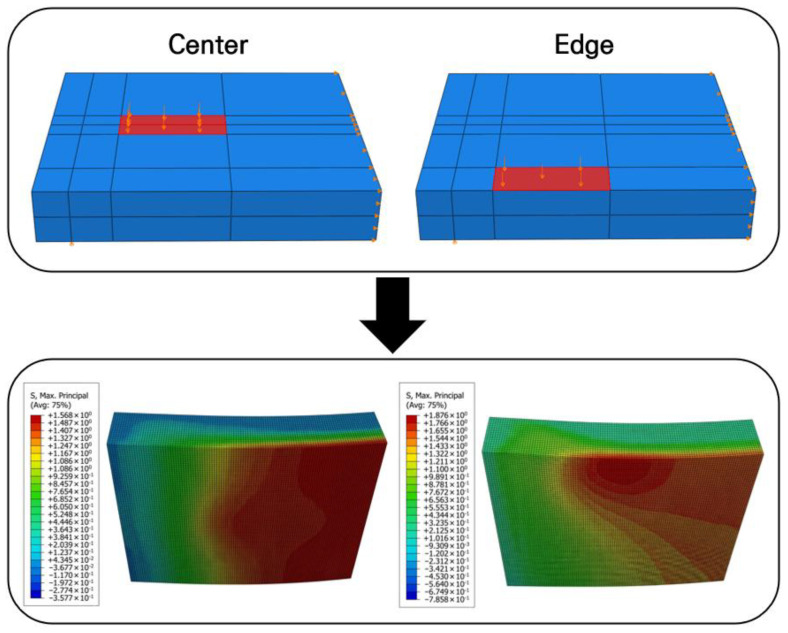
Analysis results according to loading conditions.

The numerical analysis results are shown in Figure 6. As a result of analyzing the maximum stresses according to the loading locations, a maximum principal tensile stress of 1.568 MPa occurred when the load was applied to the center of the slab, and a maximum principal tensile stress of 1.874 MPa occurred when the load was applied to the edge. These maximum tensile stresses are lower than the flexural tensile strength of the concrete slab, which is about 5 MPa [59]. Therefore, it was analyzed that no structural failure or harmful cracks would occur in the slab under the loading conditions. Based on the verified structural safety of the full-scale slab, the design of the small-scale slab was subsequently performed.

## 4. Design and Fabrication of Small-Scale Slab

### 4.1. Design Using Numerical Analysis

**Figure 7:**
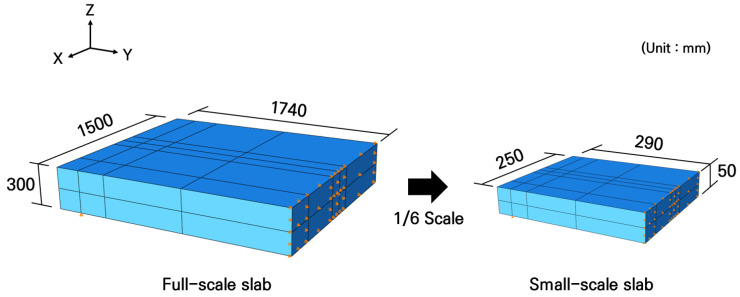
Numerical analysis models of full-scale and small-scale slabs.

To design small-scale reinforced concrete slabs, analysis models of the small-scale slabs were created using the same analysis techniques used in the full-scale slab analysis. The concrete slabs were modeled using solid elements, with dimensions of 250 mm in length, 580 mm in width, and 50 mm in thickness, by applying a dimensional reduction ratio of 1/6 to the full-scale slab dimensions. To improve the analysis efficiency, a symmetric model with a width of 290 mm was configured as shown in Figure 7, considering the load application locations and the shape of the analysis model.

When applying the reduction ratio of 1/6, the cross-sectional area of the D16 deformed steel bar applied to the full-scale slab must be reduced by a ratio of 1/36 for the small-scale slab. In this case, a steel bar with a diameter of 2.67 mm should be used. However, since the minimum size of deformed steel bars commercially available in Korea is D8 with a diameter of 8 mm, the D8 steel bars had to be used. Therefore, if the reinforcement arrangement of the full-scale slab is applied as is, a problem of excessive reinforcement ratio occurs. To compensate for this, an optimal reinforcement design was derived through numerical analyses by varying the spacing and number of steel bars so that an equivalent stress to that of the full-scale slab occurred. It should be noticed that smaller sizes of steel bar, such as 4 mm or 6 mm, are available internationally.

The material properties of the small-scale slab analysis model were assumed to be identical to those of the full-scale slab model. The mesh size of the full-scale slab analysis model was scaled down by a ratio of 1/6, defining it as 2.5 mm in each axial direction to have the same number of finite elements and nodes. The boundary and loading conditions were configured identically to the full-scale slab analysis model, and the analyses were performed by reducing the load magnitude and loading area by a ratio of 1/36.

After performing analyses according to various reinforcement arrangements using D8 steel bars in the small-scale slab analysis model, the case where stresses most similar to those of the full-scale slab was selected. Finally, the reinforcement design of the small-scale slab was conducted as presented in Table 3. As previously mentioned, it was assumed that the longitudinal steel bar ends were assumed to be simply cut. It should also be noted that the transverse steel bar ends were positioned to protrude outside of the slab during the fabrication process to facilitate the movement of the slab. The numerical analysis results of the full-scale and small-scale slabs are compared in Table 4. As shown in the table, the results obtained from the two analysis models are actually identical, thereby validating the similitude between the full-scale and small-scale slab designs.

### 4.2. Concrete Mix Design and Slab Fabrication

To fabricate the small-scale reinforced concrete slabs designed through numerical analyses, aggregate gradation and concrete mix designs were performed. In the aggregate gradation design, if the aggregate gradation of the full-scale slab is simply scaled down by a ratio of 1/6, the size of the coarse aggregate becomes excessively small, raising concerns that the inherent aggregate interlocking effect of concrete may be lost or that it may exhibit behavior similar to mortar. In such cases, the mechanical properties of the full-scale slab cannot be properly simulated, and it can negatively affect the development of the target strength. Considering this, the aggregate gradation design was performed by setting the maximum size of coarse aggregate to 13 mm and the maximum size of fine aggregate to 10 mm for the small-scale slabs. The concrete mix design was performed by applying the same mix conditions as the full-scale slabs to calculate the unit weight of water, cement, and aggregates, and the results of the aggregate gradation and concrete mix designs for the small-scale slabs are presented in Table 5.

**Figure 8:**
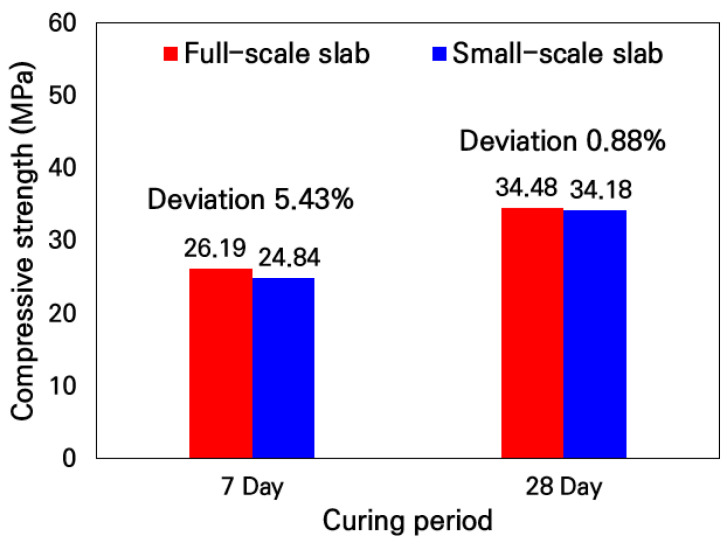
Comparison of compressive strength test results.

To verify the similarity in concrete strength between the small-scale and full-scale slabs according to the aggregate gradation and concrete mix designs, compressive strength tests were conducted in accordance with the Korean standard test method, ‘KS F 2405 Standard test method for compressive strength of concrete’ [60]. The cylindrical specimens with a diameter of 0.1 m and a height of 0.2 m were used. Eight specimens each were fabricated according to the concrete mix designs of the small-scale and full-scale slabs, and the compressive strengths were measured using four specimens each at curing periods of 7 and 28 days. As a result of the compressive strength tests, as shown in Figure 8, the compressive strengths showed a difference of about 5% on the 7th day of curing, but a difference of less than 1% occurred on the 28th day of curing. Therefore, it was analyzed that the small-scale and full-scale slabs possessed equivalent levels of concrete compressive strength, and through these experiments, it was validated that the aggregate gradation and concrete mix designs applied to the small-scale slabs were appropriate.

**Figure 9:**
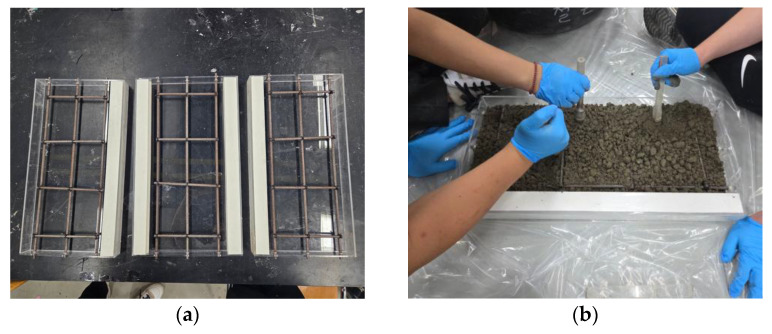

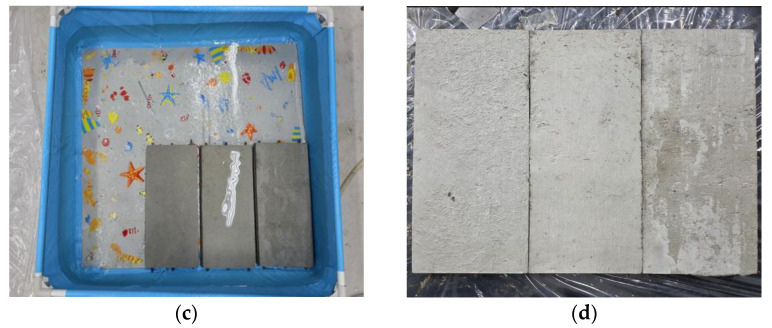
Fabrication process of small-scale slab: (**a**) formwork installation and reinforcement placement; (**b**) concrete pouring; (**c**) underwater curing; (**d**) completion.

After optimizing the design through numerical analyses and verifying the concrete mix design through compressive strength tests, the small-scale reinforced concrete slabs were fabricated. Formworks were manufactured using transparent acrylic materials to fit the designed dimensions so that the placement and compaction status of the concrete could be visually confirmed. Longitudinal and transverse steel bars were arranged inside the formwork according to the reinforcement design. Since the slab was thin, making it impossible to install general steel bar spacers, a method of fixing the steel bars by drilling holes in the formwork was applied to ensure that the steel bar locations did not change during concrete placement. Subsequently, the materials were mixed according to the determined mix ratio, the concrete was placed, and layer compaction was performed using a tamping rod. Immediately after concrete placement, surface finishing work was performed to ensure flatness, and after conducting moist curing for 24 h to secure initial strength, the formworks were removed. Thereafter, underwater curing was performed to complete three identical small-scale experimental slabs. The experimental slabs were named Slab A, Slab B, and Slab C, respectively. Figure 9 shows the major fabrication processes of the small-scale experimental slabs.

## 5. Behavior Analysis of Small-Scale Slab

The behavior of the small-scale slab is analyzed by performing load tests, and based on this, the behavior of the full-scale slab is intended to be predicted. First, a numerical analysis model was created to verify the reliability of the behavior measured in the experiments, and the structural behavior characteristics according to the variation in the elastic modulus of concrete were analyzed. Furthermore, to accurately measure the micro-behavior of the small-scale slab, experiments were performed using strain gauges of various lengths, and the optimal strain gauge was selected through comparison with the numerical analysis results. In addition, a method to accurately measure the deflection of the small-scale slab using displacement transducers was established. Subsequently, the selected measurement sensors were installed on the small-scale slabs, and load tests were performed to analyze the experimental behavior characteristics of the slabs.

### 5.1. Numerical Analysis

A numerical analysis model was created to analytically evaluate the structural behavior of the small-scale slab. The analysis model was configured with the same dimensions as the fabricated small-scale slab: 250 mm in length, 580 mm in width, and 50 mm in thickness, and was modeled using solid elements. The size and arrangement of the steel bars were modeled using wire elements, reflecting the dimensions of the design drawings. The analysis was planned to be performed considering various elastic moduli of concrete, specifically 25 GPa, 27 GPa, 27.5 GPa, and 28 GPa, so that the actual elastic modulus could be predicted by comparing the numerical analysis results with the experimental results. The Poisson’s ratio of the concrete was assumed to be 0.15. The elastic modulus and Poisson’s ratio of the steel bars were assumed to be 200 GPa and 0.3, respectively. The size of the finite element mesh was set to 2.5 mm in each axial direction, identical to the previous analysis. For the boundary conditions, the vertical displacements were constrained at the slab support locations to simulate the situation where supports are placed at the corners of the slab. For the loading condition, a load of 754.6 N was applied over a square area of 30 mm by 30 mm at the center of the slab.

**Figure 10:**
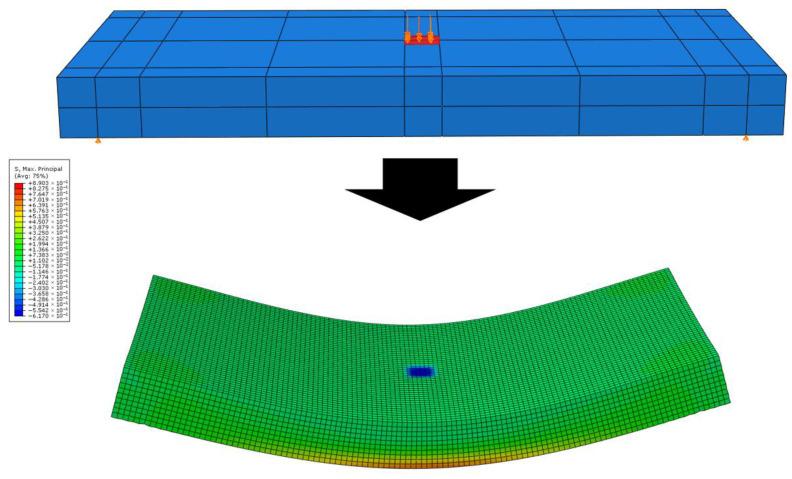
Example of numerical analysis result.

Figure 10 shows an example of the numerical analysis results, and the analysis results of the strain and deflection of the slab according to the change in the elastic modulus of concrete are presented in Table 6. These numerical analysis results will be utilized as data for the subsequent sensor selection and comparative validation with the experimental results of the small-scale slabs.

### 5.2. Selection of Measurement Sensors

To accurately evaluate the behavior of the small-scale slab, reliable strain and deflection experimental data must be obtained. Therefore, the strain gauges must be selected considering the material characteristics of concrete and the optimal specifications for displacement transducers must also be selected.

**Figure 11:**
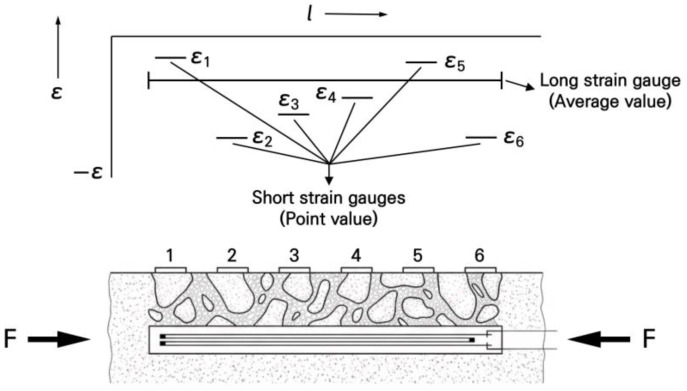
Strain difference according to strain gauge length [61].

Generally, when measuring strains on a concrete surface, it is recommended to secure a strain gauge length equivalent to approximately 4 to 5 times the maximum aggregate size to minimize errors caused by local strain variations due to the material heterogeneity between the coarse aggregates and the cement paste [61]. If the gauge length is insufficient, as shown in Figure 11, excessive local strain variations may occur depending on whether the attachment location is on an aggregate or the cement paste, raising concerns that it may not represent the average strain of the concrete member.

**Figure 12:**
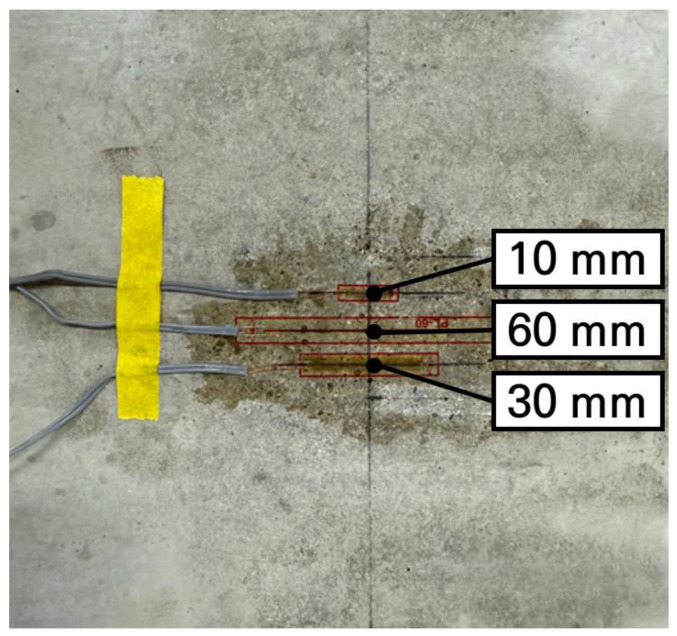
Installation of different strain gauges.

Therefore, to determine the most suitable strain gauge length for the small-scale slab, an experiment was conducted to compare the strain deviations according to the gauge length. In the experiment, as shown in Figure 12, 3 types of strain gauges were used: a 60 mm gauge commonly used for concrete members, a small 10 mm gauge considering the dimensional reduction ratio of 1/6 for the small-scale slab, and a 30 mm gauge as an intermediate size. The strain gauges used in this experiment were PL-60, PFL-10, and PFL-30 (manufactured by TML, Tokyo, Japan). Strain gauge-based measurement systems are known to provide a resolution of less than 1 micro-strain, which means they can reliably measure strain levels of about 1 micro-strain [62]. These strain gauges were attached to the centers of 3 fabricated slabs, and after performing the load tests, the measurement data were compared and analyzed. The support and loading conditions of the slabs were configured identically to the numerical analysis so that the reliability could be validated by comparing the experimental and numerical analysis results.

The slab strains measured through the load tests are presented in Table 7 for 3 experimental slabs. As shown in the table, it can be seen that even at almost the same locations of the slab, the strains are measured differently depending on the length of the strain gauge. Accordingly, as a result of analyzing the measured values by gauge length to determine which gauge provides more stable measurements, it was found that the 10 mm and 30 mm strain gauges exhibited clear differences in the measured values across the 3 slabs, with the 10 mm gauge showing the largest standard deviation. In contrast, the 60 mm strain gauge showed highly similar measured values across the 3 slabs, with a negligibly small standard deviation. As previously described, it was validated that if the gauge length is too short, it sensitively reacts to the local behaviors of the concrete aggregate and cement paste, making it difficult to stably measure the overall behavior of the concrete member. Therefore, when measuring the strain of the small-scale slab, if the gauge length is also scaled down according to the slab reduction ratio, it is difficult to obtain appropriate measurement values. Thus, it is desirable to use the strain gauges generally applied to concrete members. For this reason, 60 mm strain gauges were selected in this study.

To precisely measure the deflection behavior of the small-scale slab, the appropriate measurement range of the displacement transducer was examined based on the preceding numerical analysis results. As a result of the numerical analysis, the maximum possible deflection in the slab was predicted to be approximately 0.035 mm. This is a level that can be measured by a displacement transducer, a displacement transducer commonly utilized in concrete member experiments. Therefore, in this study, the CDP-10 (manufactured by TML, Tokyo, Japan) with a measurement length of 10 mm was selected [63]. This sensor has a rated capacity of 10 mm, an output of 5 mV/V, a non-linearity within 0.1% RO, and a sensitivity of 1000 × 10^−6^ strain/mm [63], making it a displacement transducer capable of reliably measuring micro-displacements at the level of 0.035 mm or less.

### 5.3. Strain Analysis

**Figure 13:**
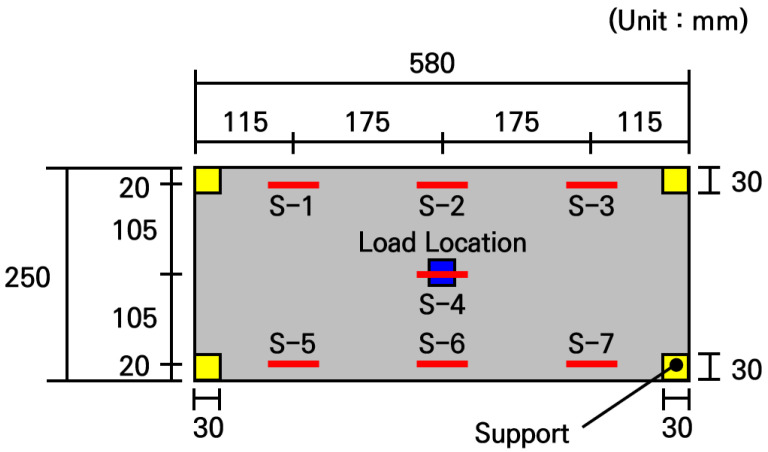
Overview of strain measurement experiment.

**Figure 14:**
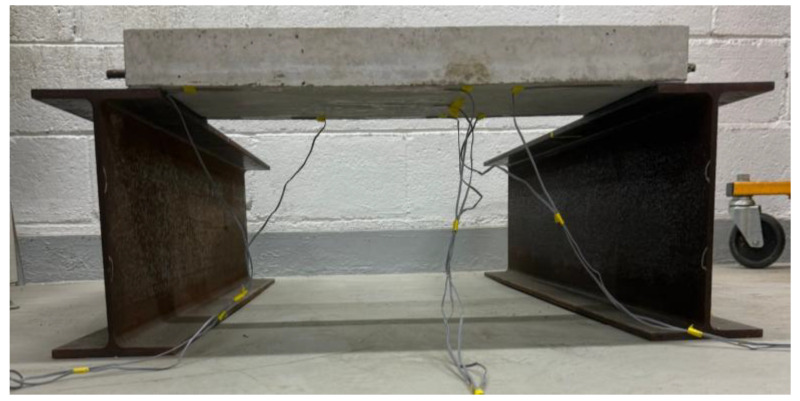
Configuration of strain measurement experimental system.

The strain measurement experiments were conducted using the fabricated small-scale slabs. Seven strain gauges were attached to the surface of the slab as shown in Figure 13, and the support conditions were applied identically to the numerical analysis model. For the loading condition, identical to the numerical analysis model, a load of 754.6 N was applied to a 30 mm by 30 mm area at the center of the slab. To ensure the reliability of the experiment, load tests were performed 3 times for each of the 3 slabs to verify the reproducibility of the measured values. In addition, to ensure structural stability during the measurement process and to secure space for installing strain gauges and displacement transducers, high-rigidity H-beams were placed under the supports to construct the experimental system as shown in Figure 14.

The experimental analysis results for each slab are presented in Table 8. In all slabs, the maximum strain occurred at the center where the load was applied, and the strain tended to decrease as the distance from the loading location increased. The results of the 3 tests for each slab were very similar, confirming that the experiments were appropriately conducted. When comparing the strains among the slabs, although there were very slight differences, the deviation was almost 0, confirming that the fabricated slabs exhibited virtually uniform structural behavior.

**Figure 15:**
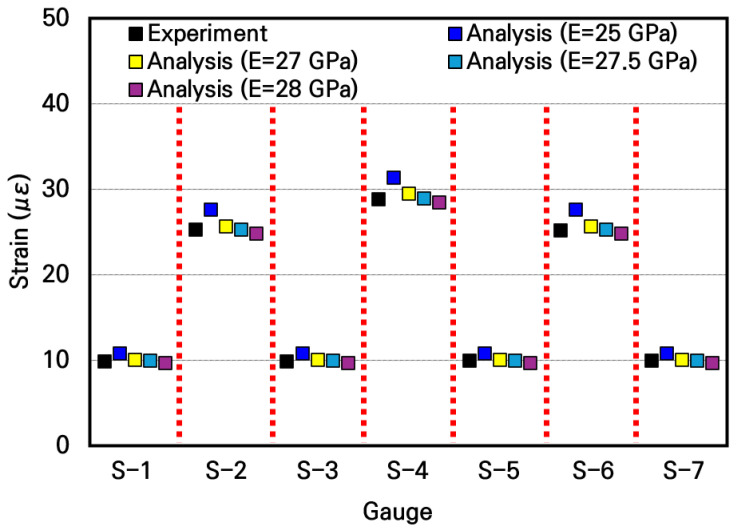
Comparison of strains between experimental and numerical analysis results.

To evaluate the elastic modulus of the small-scale slab, the measured strains were compared with the numerical analysis results according to the elastic modulus, as shown in Figure 15. The experimentally measured strains are similar to the analytical strains when the typical elastic moduli of concrete in the range of 25 GPa to 28 GPa are used in the numerical analysis. In particular, it can be seen that the measured strains are most similar to the strains of the numerical analysis model applying an elastic modulus of 27.5 GPa, with a deviation confirmed to be within approximately 1%. Furthermore, in this case, the deviation at the slab center where the maximum strain occurs was within 0.5%, meaning the measured strain practically coincided with the analytical strain. Through this, the elastic modulus of concrete of the small-scale slab can be inferred as 27.5 GPa.

### 5.4. Deflection Analysis

**Figure 16:**
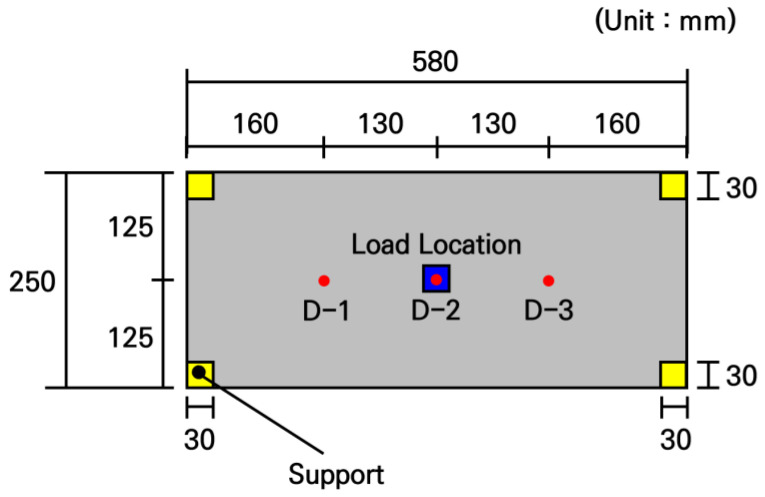
Overview of deflection measurement experiment.

**Figure 17:**
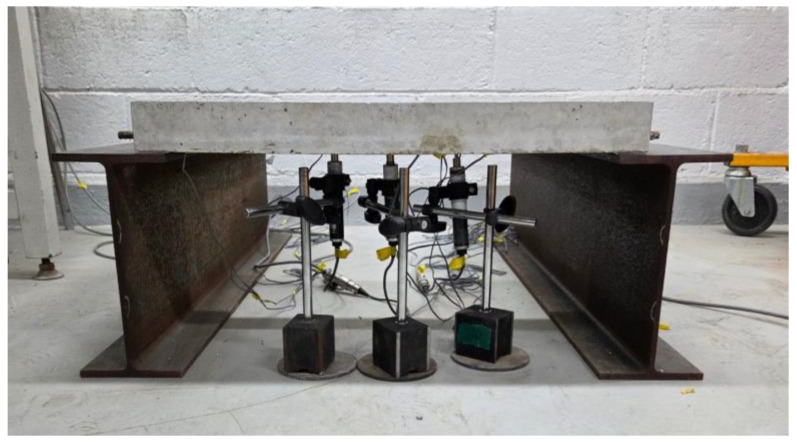
Configuration of deflection measurement experimental system.

The deflection measurement experiments were conducted using the fabricated small-scale slabs. To measure the deflection distribution of the slab under loading, a total of 3 displacement transducers were installed at the bottom center of the slab and at two other locations spaced apart from each other, as shown in Figure 16. The loading and support conditions were configured identically to the strain measurement experiment, i.e., a load of 754.6 N was applied to a 30 mm by 30 mm area at the center of the slab. As shown in Figure 17, H-beams were placed under the supports to secure installation space for the displacement transducers and measurement stability.

Under the same conditions, load tests were performed 3 times for each slab, and the experimental analysis results are presented in Table 9. In all slabs, the maximum deflection occurred at the center where the load was applied, and the deflection tended to decrease as the distance from the loading location increased. As a result of comparing the deflections among the slabs, the deviation was not significant, remaining within approximately 4%, confirming that the fabricated slabs exhibited similar structural behavior.

**Figure 18:**
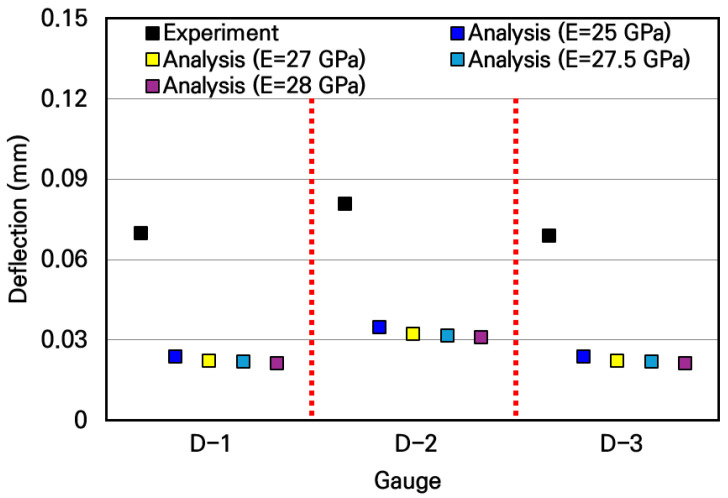
Comparison of deflections between experimental and numerical analysis results.

The experimental deflections of the small-scale slabs and the analytical deflections according to the elastic modulus of concrete were compared and the results are shown in Figure 18. As can be seen in the figure, it was observed that the experimental deflections appeared approximately 2 to 3 times larger than the analytical deflections. Since the accuracy of the numerical analysis model had been validated in the previous strain experiment, it could be expected that an unforeseen experimental error existed in this deflection measurement experiment. As a result of carefully reviewing the experimental process, the cause of the experimental error was that deflections also occurred in the flanges of the H-beams where the supports were installed. It was confirmed that in the deflection measurement experimental system shown in Figure 16 and Figure 17, the displacement transducers measured not only the deflections of the slab but also the deflections of the H-beam flanges. Therefore, since the displacements occurring in the small-scale slab experiments are extremely small, it is evident that measurements must be performed considering the micro-displacements of the surrounding structures supporting the experimental slab.

**Figure 19:**
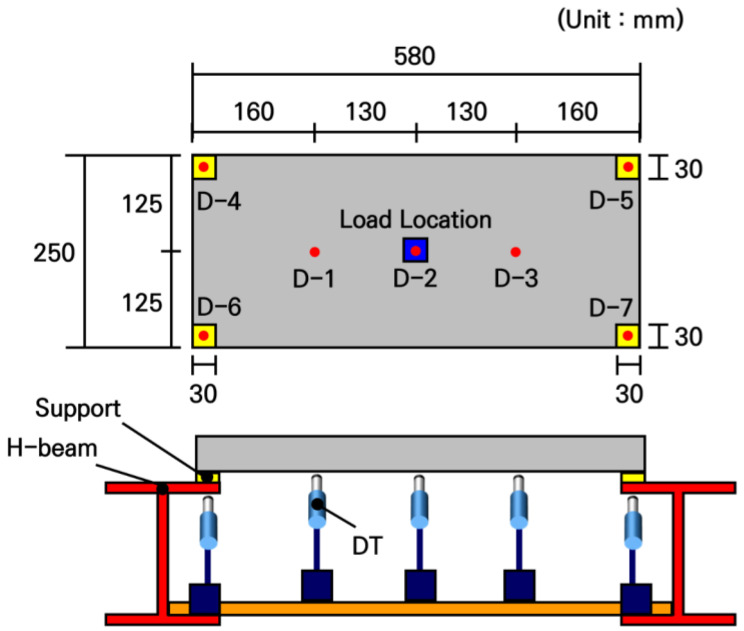
Overview of modified deflection measurement experiment.

**Figure 20:**
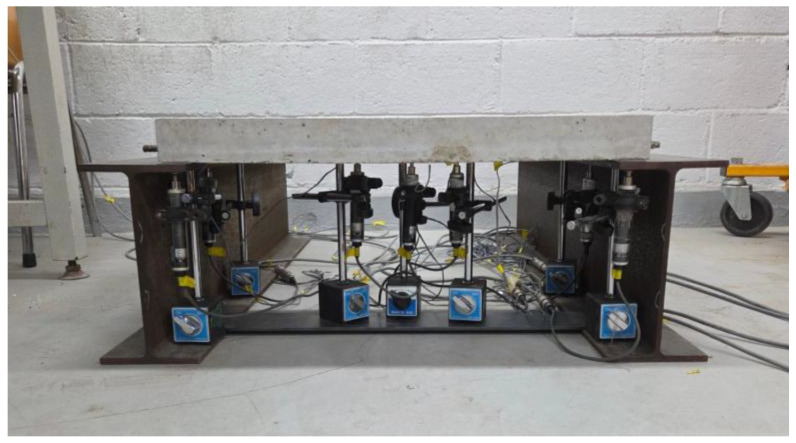
Configuration of modified deflection measurement experimental system.

Thus, to measure only the pure deflection of the slab, a measurement system capable of compensating for the displacements of the structures supporting the slab was designed, and the experiments were repeated. The schematic diagram of the modified slab deflection measurement experiment is shown in Figure 19, and the configuration of the actual deflection measurement experimental system is shown in Figure 20. The loading and support conditions were identical to those used in the previous experiments, with a live load of 754.6 N applied over a 30 mm by 30 mm area at the center of the slab. In addition to the 3 displacement transducers for deflection measurement installed under the slab, 4 more displacement transducers were installed under the H-beam flanges where the slab supports were placed, resulting in a total of 7 displacement transducers. These displacement transducers were not installed directly on the ground; instead, they were installed using a bridge designed to behave identically to the bottom flanges of the H-beams. This measurement system was configured to prevent any displacement caused by the H-beam settling into the ground from affecting the measured deflections.

The pure deflection of the slab was calculated by subtracting the settlement of the support points measured under the H-beam flanges from the displacement measured under the slab. The deflection experimental results of the small-scale slab derived through this correction process are summarized in Table 10. As shown in the table, the pure deflections of the slab, which are the corrected deflection values, can be obtained by subtracting the correction value representing the deflection of the support points from the uncorrected deflection value measured by the displacement transducers under the slab.

**Figure 21:**
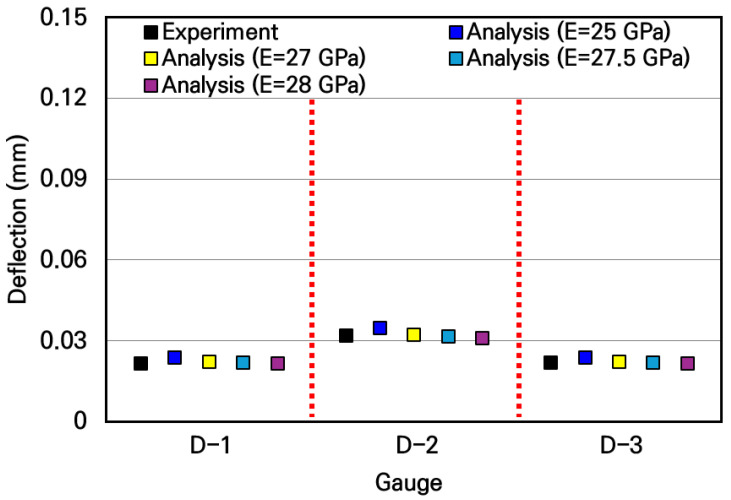
Comparison of deflections between corrected experimental and numerical analysis results.

The corrected slab deflection experimental results and the numerical analysis results were compared and analyzed, as shown in Figure 21. Similar to the previous comparative analysis results of strains, the measured deflection experimental values are similar to the analytical values when the typical elastic moduli of concrete in the range of 25 GPa to 28 GPa are used in the numerical analysis. In particular, when the slab’s elastic modulus of 27.5 GPa, obtained through the strain experiment, was used, the experimental and analytical deflections of the slab were most similar, with a deviation analyzed to be approximately 0.8%. Therefore, it was verified that the deflection of the small-scale slab can be accurately evaluated by using the modified deflection measurement experimental system.

## 6. Confirmation of Relationship Between Full-Scale and Small-Scale Slabs

To verify whether the experimental and numerical analysis results of small-scale slabs can predict the structural behavior of full-scale slabs, the numerical analysis of full-scale slabs was performed. The numerical analysis model of the full-scale slab is the same as the previously described model, and the size of the finite element mesh is configured to be six times the size converted inversely to the reduction ratio used for the small-scale slab. The elastic modulus of concrete was determined to be 27.5 GPa, based on experimental and numerical analysis results for small-scale slabs. Furthermore, the loading and support conditions were identical to those used in the small-scale slab experiments and analysis models.

The numerical analysis results for the strain of the full-scale slab are compared with the experimental and numerical analysis results for the strain of the small-scale slab and are presented in Table 11. As can be seen in the table, the numerical analysis results of the full-scale slab are very similar to the experimental results of the small-scale slab, with a maximum deviation of approximately 3.8%. Therefore, it was confirmed that the strain of a full-scale slab can be appropriately predicted using the strain experimental results of a small-scale slab.

The numerical analysis results for the deflection of the full-scale slab are compared with the experimental and numerical analysis results for the deflection of the small-scale slab and are presented in Table 12. When comparing the displacements of a full-scale slab and a small-scale slab, the dimensional reduction ratio must be taken into account. That is, the deflection of the full-scale slab must be multiplied by the reduction ratio of 1/6 and then compared to the deflection of the small-scale slab. As can be seen from the table, the analysis results of the full-scale slab are virtually identical to the experimental results of the small-scale slab. Therefore, it was confirmed that the deflection of a full-scale slab can be predicted very accurately using the deflection experimental results of a small-scale slab.

## 7. Summary and Conclusions

This study was conducted to develop a methodology for evaluating the behavior of reinforced concrete slabs used in temporary traffic bridge systems installed on uncured cement concrete pavements using small-scale experimental reinforced concrete slabs with a high-dimensional reduction ratio. A small-scale reinforced concrete slab was designed considering a dimensional reduction ratio of 1/6 and available small sizes of steel bars so that the full-scale and small-scale slabs exhibit the same behaviors in numerical analyses. The experimental small-scale slabs were manufactured by mixing concrete to ensure that the compressive strength of the small-scale slabs was similar to that of the full-scale slabs. The methods for selecting strain gauges and for installing displacement gauges were proposed to accurately measure the behavior of small-scale slabs. The experimental and numerical analysis results of the small-scale slab were consistent, and the numerical analysis results of the small-scale slab and the full-scale slab were identical, proving that the experimental analysis results of the full-scale slab can be inferred through experiments using the small-scale slab. The important findings from this study are provided as follows:

- When conducting experiments to predict the behavior of a full-scale reinforced concrete slab using a small-scale reinforced concrete slab, the size of the small-scale slab can be determined by simply applying a dimensional reduction ratio, but since it is impossible to reduce the steel bars equally, various reinforcement designs were performed using steel bars of small sizes that can actually be obtained, and then it was confirmed through numerical analyses that a reinforcement design that exhibits behavior almost similar to that of a full-scale slab can be applied to fabricate a small-scale slab.
- In small-scale reinforced concrete slabs, the size of coarse aggregates must also be reduced, so test specimens for concrete compressive strength using the concrete mix designs for full-scale and small-scale slabs must be manufactured separately, and compressive strength tests must be performed to confirm that the compressive strengths are almost identical before fabricating small-scale slabs.
- When conducting experiments using small-scale reinforced concrete slabs, it was verified that a strain gauge of a size generally used in concrete member experiments should be used. If the concrete strain gauge with a reduced length is used, errors may occur because it could measure local strain in the aggregate or cement paste.
- When measuring the deflection of small-scale reinforced concrete slabs, even small displacements of the supporting structure can affect the measured values because the slab deflection is extremely small. Therefore, it was confirmed that in order to accurately measure the pure deflection of the slab, the measurement values must be corrected by measuring not only the deflection of the slab but also the displacement of the supporting structure.
- Comparing the measured behavior of the small-scale slab with the numerical analysis results, it was confirmed that the same behavior was observed. Therefore, the experimental results and numerical analysis results of the small-scale slab were consistent, and the numerical analysis results of the small-scale slab and the full-scale slab were identical, proving that the experimental results of the full-scale slab can be inferred through experiments using the small-scale slab.

This study confirmed that if small-scale reinforced concrete slabs are designed and manufactured to appropriately reflect the characteristics of full-scale reinforced concrete slabs, the behavior of full-scale slabs can be evaluated conveniently and economically through experiments using small-scale experimental slabs. Based on the results of this study, it may be possible to appropriately predict the behavior of full-scale reinforced concrete structures using scaled-down structures not only in reinforced concrete slabs but also in other types of reinforced concrete structures. The reinforced concrete slabs considered in this study were analyzed based on the range of the linear elastic region prior to cracking, and it should be noted that the similarity between full-scale and small-scale reinforced concrete slabs regarding behavior after cracking requires verification through further research.

## Figures and Tables

**Table 1:** Reinforcement design of full-scale slab.

Category	Steel Bar Diameter (mm)	Steel Bar Length (mm)	Steel Bar Spacing (mm)	Number of Steel Bars	Concrete Cover (mm)
Longitudinal steel bar	16	1350	330	11	75
Transverse steel bar	16	3330	225	7	75
Reinforcement design drawing	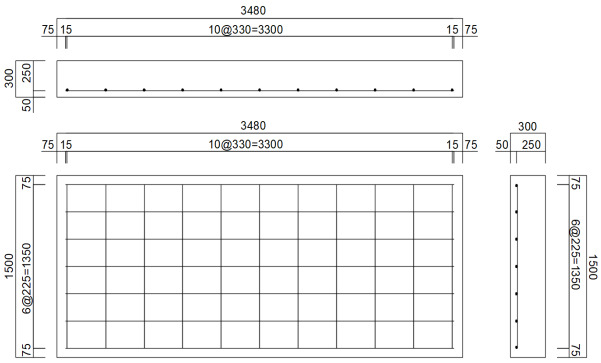				

**Table 2:** Aggregate gradation and concrete mix design of full-scale slab.

Aggregate Gradation					
Coarse Aggregate			Fine Aggregate		
Aggregate Size (mm)	Passing Percentage (%)	Fraction by Size Range (%)	Aggregate Size (mm)	Passing Percentage (%)	Fraction by Size Range (%)
25	100.0	0.0	10	100.0	0.0
20	95.0	5.0	5	97.5	2.5
10	37.5	57.5	2.5	90.0	7.5
5	5.0	32.5	1.2	70.0	20.0
2.5	2.5	2.5	0.6	45.0	25.0
2.5>	0.0	2.5	0.3	22.5	22.5
			0.1	8.5	14.0
			0.1>	0.0	8.5
**Concrete mix design**					
Volume (m^3^)	Water (kg)	Cement (kg)	Coarse aggregate (kg)	Fine aggregate (kg)	
1.566	258	646	1074	1140	

**Table 3:** Reinforcement design of small-scale slab.

Category	Steel Bar Diameter (mm)	Steel Bar Length (mm)	Steel Bar Spacing (mm)	Number of Steel Bars	Concrete Cover (mm)
Longitudinal steel bar	8	220	150	4	15
Transverse steel bar	8	550	110	3	15
Reinforcement design drawing	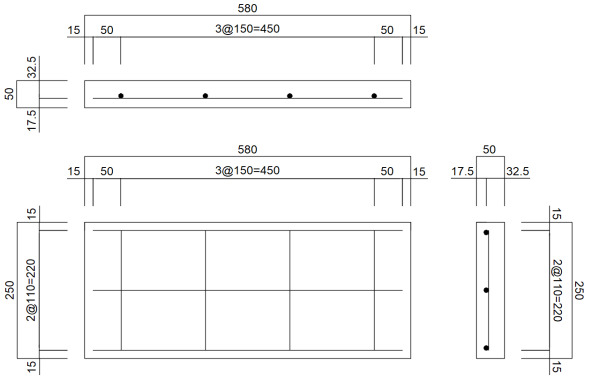				

**Table 4:** Comparison of numerical analysis results.

Load Application Location	Maximum Principal Tensile Stress (MPa)		Difference (%)
	Full-Scale Slab	Small-Scale Slab	
Edge	1.874	1.870	0.2
Center	1.568	1.548	1.3

**Table 5:** Aggregate gradation and concrete mix design of small-scale slab.

Aggregate Gradation					
Coarse Aggregate			Fine Aggregate		
Aggregate Size (mm)	Passing Percentage (%)	Fraction by Size Range (%)	Aggregate Size (mm)	Passing Percentage (%)	Fraction by Size Range (%)
20	100	0	10	100	0
13	95	5	5	97.5	2.5
10	55	40	2.5	90	7.5
5	7.5	47.5	1.2	70	20
2.5	2.5	5	0.6	45	25
2.5>	0	2.5	0.3	22.5	22.5
			0.1	8.5	14
			0.1>	0	8.5
**Concrete mix design**					
Volume (m^3^)	Water (kg)	Cement (kg)	Coarse aggregate (kg)	Fine aggregate (kg)	
0.00725	1.20	2.99	4.86	5.38	

**Table 6:** Numerical analysis results.

Elastic Modulus of Concrete (GPa)	Strain (με)	Deflection (mm)
25.0	31.348	0.0349
27.0	30.389	0.0324
27.5	30.254	0.0318
28.0	30.138	0.0312

**Table 7:** Slab strain measurement results according to strain gauge length.

Slab	Gauge Length (mm)	Measured Strain (με)	Gauge Length (mm)	Slab	Measured Strain (με)	Standard Deviation (με)
A	10	30	10	A	30	3.9
	30	27		B	38	
	60	31		C	34	
B	10	38	30	A	27	3.2
	30	33		B	33	
	60	30		C	31	
C	10	34	60	A	31	0.3
	30	31		B	30	
	60	31		C	31	

**Table 8:** Strain measurement experimental results.

Specimen	Test Number	Strain at Each Gauge Location (με)						
		S-1	S-2	S-3	S-4	S-5	S-6	S-7
Slab A	1	10	25	10	29	10	25	10
	2	10	25	10	29	10	25	10
	3	9	25	10	28	10	25	10
	Average	10	25	10	29	10	25	10
Slab B	1	10	26	10	29	10	25	10
	2	10	25	10	29	10	26	10
	3	10	25	10	29	10	25	10
	Average	10	25	10	29	10	25	10
Slab C	1	10	25	10	28	10	25	10
	2	10	25	10	29	10	25	10
	3	10	25	10	28	10	25	10
	Average	10	25	10	29	10	25	10
Average strain		10	25	10	29	10	25	10

**Table 9:** Deflection measurement experimental results.

Specimen	Test Number	Deflection at Each Displacement Transducer Location (mm)		
		D-1	D-2	D-3
Slab A	1	0.071	0.083	0.072
	2	0.073	0.085	0.072
	3	0.072	0.081	0.070
	Average	0.072	0.083	0.071
Slab B	1	0.070	0.081	0.071
	2	0.069	0.081	0.069
	3	0.070	0.083	0.071
	Average	0.070	0.082	0.070
Slab C	1	0.069	0.081	0.068
	2	0.068	0.080	0.066
	3	0.066	0.079	0.066
	Average	0.068	0.080	0.067
Average Deflection		0.070	0.082	0.069

**Table 10:** Modified deflection measurement experimental results.

Specimen	Test Number	Deflection at Each Displacement Transducer Location (mm)						
		Correction Value	Uncorrected			Corrected		
			D-1	D-2	D-3	D-1	D-2	D-3
Slab A	1	0.050	0.069	0.081	0.072	0.019	0.031	0.022
	2	0.051	0.066	0.080	0.073	0.015	0.029	0.021
	3	0.050	0.072	0.084	0.070	0.022	0.034	0.021
	Average	0.050	0.069	0.082	0.072	0.019	0.032	0.021
Slab B	1	0.049	0.072	0.080	0.071	0.023	0.031	0.021
	2	0.049	0.072	0.078	0.068	0.023	0.029	0.019
	3	0.052	0.072	0.081	0.069	0.021	0.029	0.017
	Average	0.050	0.072	0.080	0.069	0.022	0.029	0.019
Slab C	1	0.047	0.071	0.083	0.071	0.023	0.035	0.023
	2	0.048	0.073	0.082	0.073	0.026	0.035	0.026
	3	0.047	0.072	0.082	0.074	0.026	0.036	0.027
	Average	0.047	0.072	0.082	0.073	0.025	0.035	0.025
Average Deflection		0.049	0.071	0.081	0.071	0.022	0.032	0.022

**Table 11:** Strain comparison between full-scale and small-scale slabs.

Case	Strain at Each Gauge Location (με)						
	S-1	S-2	S-3	S-4	S-5	S-6	S-7
Small-scale slab (Experiment)	10	25	10	29	10	25	10
Small-scale slab (Numerical analysis)	10	25	10	29	10	25	10
Full-scale slab (Numerical analysis)	10	26	10	28	10	26	10
Difference (%)	0	3.8	0	3.6	0	3.8	0

**Table 12:** Deflection comparison between full-scale and small-scale slabs.

Case		Deflection at Each Gauge Location (mm)		
		D-1	D-2	D-3
Small-scale slab (Experiment)		0.022	0.032	0.022
Small-scale slab (Numerical analysis)		0.022	0.032	0.022
Full-scale slab (Numerical analysis)	Unscaled	0.132	0.191	0.132
	Scaled down	0.022	0.032	0.022
Difference (%)		0	0	0

## Data Availability

The original contributions presented in this study are included in the article. Further inquiries can be directed to the corresponding author.
